# An Optimal Internet of Things-Driven Intelligent Decision-Making System for Real-Time Fishpond Water Quality Monitoring and Species Survival

**DOI:** 10.3390/s24237842

**Published:** 2024-12-08

**Authors:** Saima Kanwal, Muhammad Abdullah, Sahil Kumar, Saqib Arshad, Muhammad Shahroz, Dawei Zhang, Dileep Kumar

**Affiliations:** 1 Engineering Research Centre of Optical Instrument and Systems, Ministry of Education and Shanghai Key Lab of Modern Optical System, University of Shanghai for Science and Technology, No. 516 Jun Gong Road, Shanghai 200093, China; 2 Department of Biomedical Engineering, School of Health Science and Engineering, University of Shanghai for Science and Technology, No. 516 Jun Gong Road, Shanghai 200093, China; 3 Department of Software Engineering, The Islamia University of Bahawalpur, Bahawalpur 63100, Pakistan; 4 Department of Computer Science, Shaheed Zulfikar Ali Bhutto Institute of Science and Technology (SZABIST), University Larkana Campus, Larkana 77150, Pakistan; 5 Faculty of Engineering, The Islamia University of Bahawalpur, Bahawalpur 63100, Pakistan

**Keywords:** fishpond, IoT cloud platform, machine learning, sensors, water quality parameters, climate-induced variability

## Abstract

Smart fish farming faces critical challenges in achieving comprehensive automation, real-time decision-making, and adaptability to diverse environmental conditions and multi-species aquaculture. This study presents a novel Internet of Things (IoT)-driven intelligent decision-making system that dynamically monitors and optimizes water quality parameters to enhance fish survival rates across various regions and species setups. The system integrates advanced sensors connected to an ESP32 microcontroller, continuously monitoring key water parameters such as pH, temperature, and turbidity which are increasingly affected by climate-induced variability. A custom-built dataset comprising 43,459 records, covering ten distinct fish species across diverse pond environments, was meticulously curated. The data were stored as a comma-separated values (CSV) file on the IoT cloud platform ThingSpeak and synchronized with Firebase, enabling seamless remote access, control, and real-time updates. Advanced machine learning techniques, with feature transformation and balancing, were applied to preprocess the dataset, which includes water quality metrics and species-specific parameters. Multiple algorithms were trained and evaluated, with the Decision Tree classifier emerging as the optimal model, achieving remarkable performance metrics: 99.8% accuracy, precision, recall, and F1-score, a 99.6% Matthews Correlation Coefficient (MCC), and the highest Area Under the Curve (AUC) score for multi-class classification. Our framework’s capability to manage complex, multi-species fishpond environments was validated across diverse setups, showcasing its potential to transform fish farming practices by ensuring sustainable climate-adaptive management through real-time water quality optimization. This study marks a significant step forward in climate-smart aquaculture, contributing to enhanced fish health, survival, and yield while mitigating the risks posed by climate change on aquatic ecosystems.

## 1. Introduction

Aquaculture, the controlled cultivation of aquatic organisms, plays a vital role in meeting the increasing global demand for seafood. This demand is driven by population growth, the depletion of wild fish stocks, and evolving consumer preferences for healthier, sustainable food sources [1]. However, traditional fish farming methods face several challenges, including labor-intensive monitoring, high operational costs, and the unpredictable nature of water quality fluctuations, which can lead to fish diseases and sudden mortalities. Such incidents not only impact fish survival but also increase expenses related to disease control and waste management. Given that the physical, chemical, and biological properties of water are crucial to fish health and growth, there is an urgent need for continuous, accurate water quality monitoring to improve aquaculture productivity and sustainability.

Recent advances in technology, particularly the integration of the Internet of Things (IoT) and machine learning (ML), have significantly enhanced the field of aquaculture management [2]. IoT systems, with their ability to monitor water quality in real time, have transformed aquaculture by enabling the precise and automated control of key water quality parameters such as pH, temperature, dissolved oxygen, turbidity, and salinity. By using IoT sensors, fish farmers can collect critical data remotely, facilitating real-time decision-making to maintain optimal conditions for fish survival and growth. Concurrently, ML algorithms allow the analysis of large datasets, revealing patterns that inform better management strategies—such as improved feeding practices, early disease detection, and resource optimization—while reducing human intervention. The integration of IoT and ML not only enhances operational efficiency and boosts yields but also promotes sustainability by reducing waste, conserving energy, and increasing the resilience of aquaculture systems. Various studies [3,4,5,6,7,8,9,10,11,12,13] have highlighted the benefits of these technologies, with some focusing on monitoring and surveillance, while others concentrate on recommending suitable fish species.

Numerous studies have explored the application of IoT and ML in aquaculture. For example, Chiu et al. [10] developed an IoT-based aquaculture monitoring and control system that collects and analyzes real-time water quality data, incorporating a deep learning model to predict the growth of California bass based on water quality parameters. Their approach, however, focused primarily on optimizing feeding rather than recommending appropriate fish species. Similarly, Niswar et al. [14] introduced an IoT-based water quality monitoring system for crab farming, employing Message Queuing Telemetry Transport (MQTT) and Long Range (LoRa)-based wireless sensors to enhance the survival of softshell crabs. Despite its remote monitoring capabilities, its system lacks advanced decision-making capabilities. Other research, such as that by Pikulins et al. [15] created a self-sustaining water quality monitoring system (WQMS) with hardware and software for data collection, storage, and control in remote pond environments. However, it does not incorporate automated decision support. Similarly, Murad et al. [16] designed a cost-effective, solar-powered aquaponics system that includes sensors, pumps, and GSM communication to monitor water parameters and alert users to abnormalities via text messages. While functional, it does not feature advanced intelligent components. Abinaya et al. [17] proposed an IoT-based system using sensors, Arduino, and GSM modules to monitor water quality parameters such as pH, temperature, and ammonia, triggering corrective actions when deviations occur. However, this system also lacks intelligent decision-making capabilities. Islam and Hemal et al. [18,19] developed an automated system to predict suitable fish species based on environmental parameters, achieving 88.48% and 94% accuracy using machine learning algorithms on a real-time pond water dataset [6,10]. However, the use of a Random Forest (RF) and ensemble model may not be the most practical or economical choice for fish survival prediction in aquaculture. It also failed to address the model’s validity across different fish environments in various regions, overlooking critical factors that could significantly impact the accuracy and generalizability of the model. A more balanced approach that considers both predictive performance and implementation feasibility would better serve the industry.

Furthermore, Huan et al. [20] introduced a narrow-band IoT framework for the real-time monitoring of water quality in pond aquaculture, focusing on temperature, pH, and dissolved oxygen [21]. While IoT configurations vary from basic systems with pH and temperature sensors [22] ] to more advanced systems incorporating sensors for ammonia, dissolved oxygen, and salinity [23], this study exposes a significant gap in the integration of automated decision-support features within IoT-based aquaculture systems. Many existing frameworks [3,4,6] are not equipped to deliver intelligent, real-time recommendations for fish farming based on dynamic water quality data.

Most of the existing studies [4,10,11,15] on smart fish farming largely fail to address key challenges such as full automation, real-time decision-making, adaptability to diverse environments, and sustainability. Many rely on manual monitoring or utilize a limited range of sensors that focus only on basic water quality parameters, overlooking critical factors like turbidity, which significantly affect fish health. Moreover, no prior study, to our knowledge, has developed an optimal model capable of adapting to varying environments and multi-species setups. This lack of intelligent, adaptable decision-making systems limits the applicability of current research to real-world scenarios where diverse species are farmed under dynamic environmental conditions. As a result, existing approaches fall short of maximizing productivity and supporting sustainable aquaculture practices.

In response to these gaps, we present a novel, automatic, IoT-driven intelligent decision-making system that optimally adapts to diverse environments and manages the survival of multiple species across regions, while accurately accounting for key water quality parameters. Our system combines IoT sensors with advanced ML algorithms to monitor and manage key water quality parameters including pH, temperature, and turbidity, for a variety of fish species.

The custom-built dataset of 43,459 samples captures critical water quality parameters and detailed observations of ten distinct fish species: Grass Carp (GC), Silver Carp (SC), Singhara (SG), Pangasius (PG), Common Carp (CC), Tilapia (TP), Rohu (RH), Mrigal (MR), Catla (CT), and Koi (KO). To ensure exceptional data quality and reliability, rigorous preprocessing steps were applied, including outlier detection and removal to address anomalies, feature scaling for standardization, Gaussian filtering to minimize noise, encoding for categorical variables, and data balancing to resolve class imbalances. This robustly preprocessed dataset forms a solid foundation for accurate, scalable, and impactful analysis.

We trained and evaluated several algorithms, employing GridSearchCV for systematic hyperparameter optimization to fine-tune model parameters. These included a custom ensemble model, Random Forest (RF), Support Vector Machine (SVM), Decision Tree (DT), K-Nearest Neighbors (KNN), Logistic Regression (LR), Single-Layer Perceptron (SLP), Multilayer Perceptron (MLP), and ensemble techniques such as Bagging, Boosting, and Stacking. Among these, the optimized Decision Tree (DT) model delivered superior performance across key metrics, including accuracy (ACC), precision (Pre), recall (Rec), F1-score, and Matthews Correlation Coefficient (MCC), achieving the highest AUC score for multi-class classification.

This advanced system achieves high accuracy in predicting water quality and fish survival in fishpond environments while demonstrating adaptability to diverse species setups and regions. To ensure robustness and generalizability, the model was rigorously tested on varied data sources, confirming its applicability beyond specific datasets to diverse aquaculture contexts. By integrating real-time monitoring with intelligent decision-making, this scalable and cost-effective solution offers a reliable and sustainable approach to data-driven fish farming practices, addressing critical challenges in the aquaculture industry.

## 2. Experimental Setup and Methods

**Figure 1:**
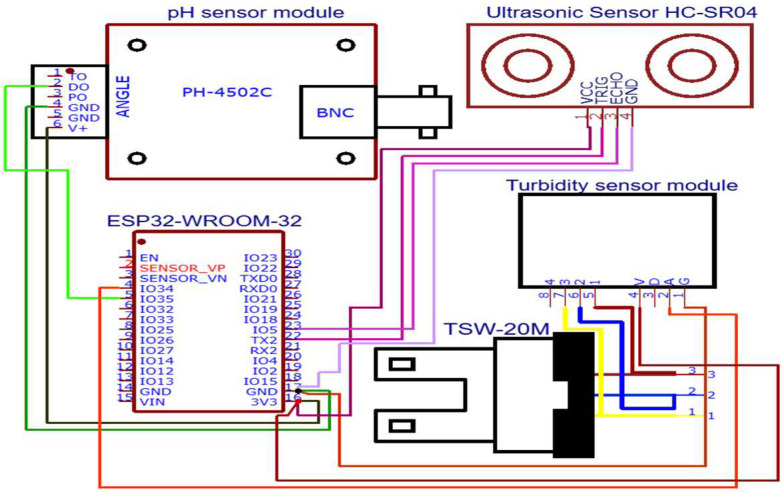
System design and prototype architecture for IoT-driven fishpond water quality monitoring. The diagram illustrates the integration of pH, temperature, turbidity, and ultrasonic sensors connected to the ESP32 microcontroller, enabling real-time data collection, transmission, and storage on the ThingSpeak IoT cloud platform.

The experimental setup for the proposed intelligent decision-making system in aquaculture is centered around a robust IoT-based architecture designed to monitor and manage fishpond environments. The system utilizes a network of strategically placed sensors to gather critical environmental data, ensuring the optimal conditions necessary for fish health and survival. These sensors include a pH sensor (Diymore PH-4502C) to measure the acidity levels, a turbidity sensor (TSW-20M) to assess water clarity, a temperature sensor (DS18B20) to track water temperature, and an ultrasonic sensor (HC-SR04) to monitor water depth. The sensors are connected to an ESP32 microcontroller, which collects, processes, and transmits the data wirelessly. Once the sensor data are gathered, the microcontroller is responsible for monitoring pH, temperature, and turbidity, which are subsequently stored as a CSV file on the IoT cloud platform ThingSpeak. Additionally, data are synchronized and stored on Firebase, a real-time database that offers enhanced flexibility and control, ensuring seamless data integration and continuous updates. The system allows remote access to real-time data and visualizations via web browsers or mobile devices, providing a convenient and user-friendly interface for monitoring pond conditions. The system is programmed using Arduino IDE 2.3.2, which simplifies code development, debugging, and uploading to the ESP32. The code is written in C++ or embedded C to ensure the efficient handling of sensor data and communication protocols. A detailed circuit diagram of the system is presented in Figure 1.

### 2.1. Selection and Analysis of Key Water Quality Parameters

This study focused on three critical water quality parameters—pH, temperature, and turbidity—due to their direct impact on fish health and their essential role in aquaculture water quality assessment. These parameters provide vital insights into metabolic activity, environmental stress, and water clarity while ensuring cost-effective system design by avoiding the complexity of additional sensors for parameters like BOD, COD, and DO. The six ponds selected represent diverse geographical and climatic conditions, with optimal ranges of pH (6.5–8.5), temperature (18–32 °C), and turbidity (0–25 NTU), ensuring system adaptability and robustness. Table 1 summarizes these standard reference values based on established aquaculture guidelines [24].

- Temperature affects critical factors like dissolved oxygen levels and electrical conductivity. Even slight temperature shifts of just 5 °C can cause severe physiological stress or mortality in aquatic organisms by altering metabolic rates and oxygen solubility.
- pH, which measures the concentration of hydrogen ions, indicates water acidity or alkalinity. Maintaining a pH range between 6.5 and 9 is essential for healthy aquaculture environments, as deviations from this range can disrupt enzymatic activity, metabolic processes, and overall fish health. Extreme pH levels may negatively affect fish growth, reproduction, and survival.
- Turbidity refers to the number of suspended particles in water, influencing light penetration and photosynthesis. High turbidity, caused by factors like sediment runoff or algal blooms, reduces light availability, which can alter fish behavior, especially their feeding patterns.

**Figure 2:**
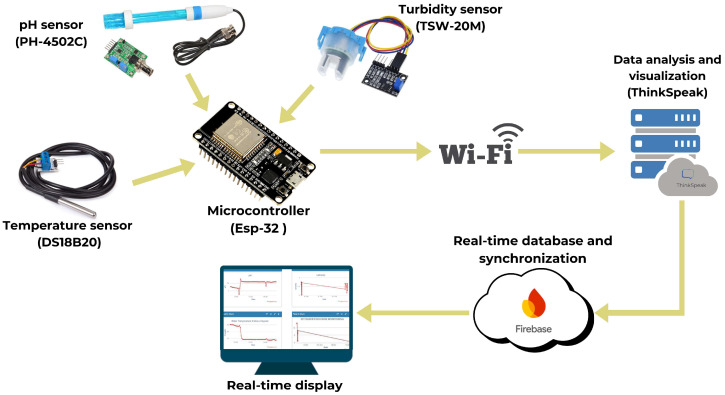
Integrated sensor array for real-time water quality analysis, featuring a pH sensor for accurate acidity and alkalinity measurements, a temperature sensor for precise thermal monitoring across a broad range, and a turbidity sensor for the effective assessment of water clarity. These components, integrated within an IoT-driven framework, ensure continuous, reliable data collection critical for adaptive aquaculture management.

As illustrated in Figure 2, the integration of the sensor array within an IoT-driven framework for real-time analysis is pivotal in providing continuous monitoring of vital water quality parameters. The temperature sensor measures from −55 °C to +125 °C with an accuracy of ±0.5 °C between −10 °C and +85 °C, ensuring reliability in harsh conditions. The pH sensor covers a range from 0 to 14 with an accuracy of ±0.1 pH and operates between 0 °C and 80 °C, providing consistent pH readings for optimal water conditions. The turbidity sensor detects water clarity from 0 to 1000 NTU with an accuracy of ±2% or ±2 NTU, functioning within a temperature range of 0 °C to 50 °C and offering both analog and digital outputs for seamless integration into monitoring systems.

### 2.2. Dataset Collection and Preprocessing

**Figure 3:**
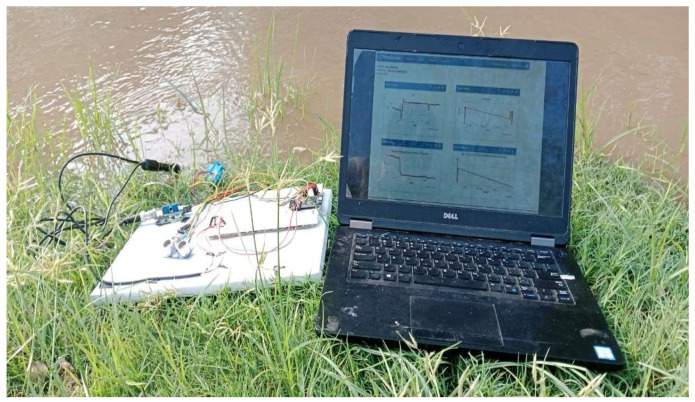
The data collection process for real-time water quality monitoring across six ponds, conducted over 157 days from January to June, during which extreme weather changes occurred. Measurements were taken every 4 h (6:00 AM, 10:00 AM, 2:00 PM, 6:00 PM, 10:00 PM, and 2:00 AM) to capture critical parameters for adaptive aquaculture management.

The real-time pond water dataset was meticulously collected over 157 days, from January to June, across six distinct ponds in various regions of Pakistan. During this time, extreme weather changes occurred, providing a diverse range of environmental conditions. Daily measurements were systematically taken every 4 h (at 6:00 AM, 10:00 AM, 2:00 PM, 6:00 PM, 10:00 PM, and 2:00 AM) to capture the fluctuations in water quality parameters throughout the day, ensuring the comprehensive data coverage of these environmental variations. This structured approach enhances both the transparency and robustness of our data collection process, allowing for a detailed analysis of water quality dynamics over time, as illustrated in Figure 3.

This comprehensive dataset comprises 43,459 records organized into four features, each representing key measured parameters and the observed fish species. The dataset includes three independent variables—pH, temperature, and turbidity—which serve as essential indicators of water quality. The pH variable has 39 unique values, the temperature has 213, and turbidity has 102, highlighting considerable variability in water quality that may significantly affect the farming suitability of different fish species. The “fish” column is designated as the target variable, capturing detailed observations of ten distinct species: GC (9481), SC (6895), SG (5771), PG (4905), CC (4195), TP (4071), RH (4066), MR (2277), CT (1043), and KO (755). These species were selected due to their economic and ecological importance in aquaculture. Despite the dataset’s extensiveness, limitations regarding its size may impact generalizability. Nonetheless, the systematic collection and organization of these data into a CSV file on ThingSpeak make it a valuable resource for further research and analysis.

We conducted a comprehensive evaluation of the dataset to assess its cleanliness, consistency, and suitability for analysis. To optimize the dataset, we applied a rigorous data preprocessing pipeline to maximize dataset quality and integrity. This process began with data visualization to gain preliminary insights, followed by an in-depth feature transformation phase. Key steps included outlier detection and removal to eliminate anomalies, feature scaling for standardized measurements, Gaussian filtering to reduce noise, encoding to handle categorical variables, and data balancing to address class imbalances effectively. These meticulously executed steps ensured an optimized dataset, providing a robust foundation for high-accuracy model training and reliable evaluation.

**Figure 4:**
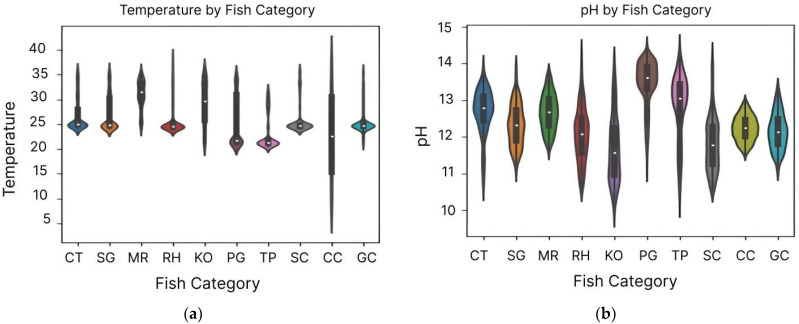

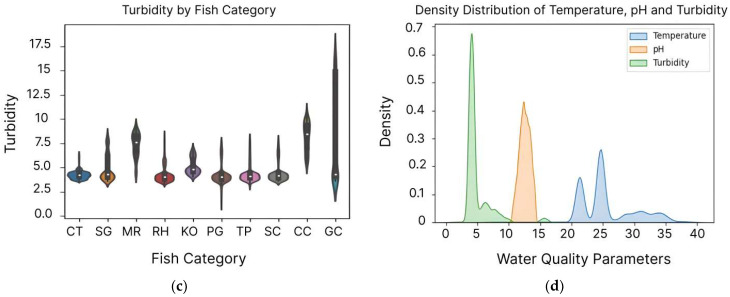
Comprehensive analysis of water quality parameters by fish category: The Violin plots (**a**–**c**) depict the distributions of temperature, pH, and turbidity, respectively, highlighting variations across fish types. The KDE plot (**d**) combines the density distributions of temperature, pH, and turbidity for a comprehensive comparison of water quality metrics.

Key variables (water quality parameters) were closely examined to detect outliers and noise. As depicted in Figure 4, the Violin plots provided an in-depth view of the distribution of individual water parameters, while the Kernel Density Estimate (KDE) plots illustrated the densities of continuous variables. The initial analysis identified irregularities, such as sharp peaks and skewed distributions, which indicated the presence of outliers and random noise. These observations guided the data cleaning process, addressing inconsistencies in larger fish categories while maintaining the integrity of smaller classes.

To effectively manage outliers and noise in the dataset, we employed Interquartile Range (IQR) filtering to remove extreme values, particularly within the larger fish categories. Outliers were defined as values exceeding 1.5 times the IQR and were removed only from fish classes with more than 1000 instances, ensuring that smaller classes remained unaffected by this process.

We normalized continuous variables through the standard scaling method, maintaining feature values within a consistent range. This scaling technique is especially beneficial for algorithms sensitive to scale variations.

After cleaning the data, a Gaussian filter was applied to the temperature, pH, and turbidity features to further reduce noise. This smoothing technique enhanced data quality and improved the performance of subsequent analyses and modeling tasks.

As the “fish” column contained categorical data, we applied encoding techniques to convert these categorical labels into numerical formats suitable for optimal multiple algorithms. Specifically, we used one-hot encoding to create binary columns for each fish species, allowing their incorporation into the model without introducing any ordinal bias.

**Figure 5:**
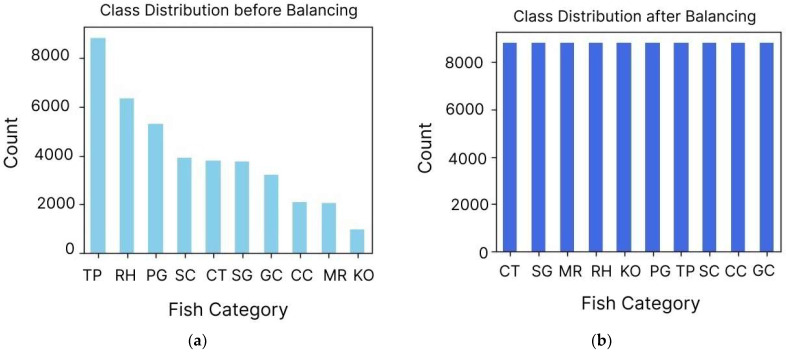
The distribution of fish species in the dataset before and after applying SMOTE. (**a**) The distribution of fish species class imbalances, with certain species being underrepresented. (**b**) A balanced representation across all fish species, which enhances the dataset’s suitability for training machine learning models.

Finally, to address the class imbalance, we identified disparities in the distribution of records across various fish categories and applied the Synthetic Minority Oversampling Technique (SMOTE) [25]. SMOTE generates synthetic samples for underrepresented classes by interpolating between existing samples, thereby balancing class distributions and reducing model bias toward majority classes. After implementing SMOTE, we verified the balanced representation of fish species, as illustrated in Figure 5.

### 2.3. Selection of Optimal Model for IoT-Driven Intelligent Decision-Making System

We analyzed several machine learning models, employing GridSearchCV for systematic hyperparameter optimization to fine-tune model parameters. This approach involved an exhaustive search over a predefined grid of hyperparameters to identify the best combinations, aiming to maximize predictive accuracy and robustness in classification tasks.

To comprehensively evaluate the performance of each model and its ability to address class imbalance, we assessed key metrics such as accuracy, precision, recall, F1-score, and Matthews Correlation Coefficient (MCC). After applying SMOTE, we observed a substantial improvement in the model’s ability to classify minority classes, particularly with notable gains in recall and F1-score, demonstrating SMOTE’s effectiveness in enhancing the identification of underrepresented classes. To mitigate the risk of overfitting, we closely monitored performance using metrics like MCC and Area Under the Curve (AUC) across both training and validation sets. For validation, the dataset was divided into training and testing sets, using an 80:20 split ratio. This standard division ensured that the models were trained on sufficient data while retaining a separate test set for robust performance evaluation.

The models included Random Forest (RF), Support Vector Machine (SVM), Decision Tree (DT), K-Nearest Neighbors (KNN), Logistic Regression (LR), Single-Layer Perceptron (SLP), Multilayer Perceptron (MLP), and ensemble techniques like Bagging, Boosting, and Stacking. These models were chosen for their diverse approaches to classification, enabling a comprehensive evaluation of predictive performance and robustness in optimizing fish species recommendations based on water quality parameters.

The DT model outperformed other algorithms due to its ability to handle non-linear relationships and effectively manage imbalanced datasets, particularly when enhanced with SMOTE. Its simplicity and interpretability, combined with high precision and computational efficiency, made it well suited for real-time decision-making in aquaculture. While ensemble models like Random Forest and Gradient Boosting demonstrated strong performance, they exhibited slightly lower accuracy due to their sensitivity to noise and higher computational demands. Simpler models such as Logistic Regression were unable to capture the dataset’s complexity, resulting in significantly lower predictive accuracy. These characteristics position DT as the ideal choice for this real-time decision-making system.

To ensure that the chosen model provides the best possible performance in this task, various evaluation metrics were employed. These metrics allow for a comprehensive understanding of the model’s behavior and help in the selection of the most effective model for real-time fish farming applications. Central to this evaluation is the confusion matrix, which contrasts the model’s predictions against the actual labels. The matrix consists of four fundamental components: true positives (*TP*), false positives (*FP*), true negatives (*TN*), and false negatives (*FN*). True positives represent the correctly predicted positive instances, while false positives indicate negative instances erroneously predicted as positive. True negatives denote accurately predicted negative instances and false negatives refer to positive instances incorrectly classified as negative.

Accuracy measures the proportion of correctly predicted instances (both true positives and true negatives) out of the total predictions made. It is often used as a general indicator of model performance across all classes. In the context of predicting fish survival based on water quality parameters, accuracy measures the overall correctness of the model’s classification. It is computed as shown in Equation (1).

(1) $$\mathrm{Accuracy}\;\;=\;\;\frac{TP+TN}{TP+TN+FP+FN}$$

Precision (positive predictive value) focuses on how many of the positive survival predictions made by the model were actually correct. Precision is critical in cases where false positives (incorrect predictions of fish survival) could lead to resource misallocation in fish farming. Precision is computed using Equation (2):

(2) $$\mathrm{Precision}\;\;=\;\;\frac{TP}{TP+FP}$$

A high precision score indicates that the model is reliable in predicting fish survival, ensuring that fewer resources are wasted on species that are predicted to survive but do not. Recall (sensitivity or true positive rate) measures the model’s ability to identify all actual survival instances correctly. In fish farming, high recall is important because missing an instance where the fish could survive (false negatives) could result in unnecessary loss. Recall is computed using Equation (3):

(3) $$\mathrm{Recall}\;\;=\;\;\frac{TP}{TP+FN}$$

A high recall score ensures that the model captures most of the species that can survive, helping to maximize productivity and avoid the loss of viable fish. F1-score balances both precision and recall, providing a single metric that captures the trade-off between these two metrics. It is especially useful in the manuscript’s context, where both false positives and false negatives can impact fish farming efficiency. The F1-score is calculated as shown in Equation (4):

(4) $$F1-\mathrm{Score}\;\;=\;\;2\times \frac{\mathrm{Precision}\times \mathrm{Recall}}{\mathrm{Precision}+\mathrm{Recall}}$$

The F1-score provides a balanced measure, ensuring that the model not only predicts fish survival accurately but also avoids missing survival opportunities. Matthews Correlation Coefficient (MCC) is a comprehensive performance metric that considers all aspects of the confusion matrix (true positives, true negatives, false positives, and false negatives). MCC is particularly important, as it evaluates model performance across different species and varying water conditions, accounting for class imbalance. The MCC is calculated using Equation (5):

(5) $$\mathrm{MCC}\;\;=\;\;\frac{\left(TP\times TN)-(FP\times FN\right)}{\sqrt{(TP+FP)\times (TP+FN)\times (TN+FP)\times (TN+FN)\;}}$$

An MCC score close to 1 indicates that the model performs well across all fish species and environmental conditions, making it a robust criterion for model selection in real-time fish farming.

### 2.4. Model Validation and Generalization

The model validation process involves three different datasets across different regions and includes publicly available datasets, as well as a custom-built dataset collected from the fish pond setups. Each dataset contains various species of fish with associated water quality parameters. The optimized Decision Tree (DT) model, fine-tuned with GridSearch and SMOTE, is applied to each dataset. The model is designed to classify different fish species based on the monitored environmental factors, capturing the unique characteristics of each species across datasets. The model’s consistent performance across diverse datasets demonstrates its strong generalization capability. This means that the model can accurately classify species in various fish ponds, even with differences in environmental conditions and species compositions, making it highly applicable to new datasets in similar contexts.

## 3. Results and Discussion

### 3.1. Optimal Model Performance Evaluation in Smart Fish Farming

**Figure 6:**
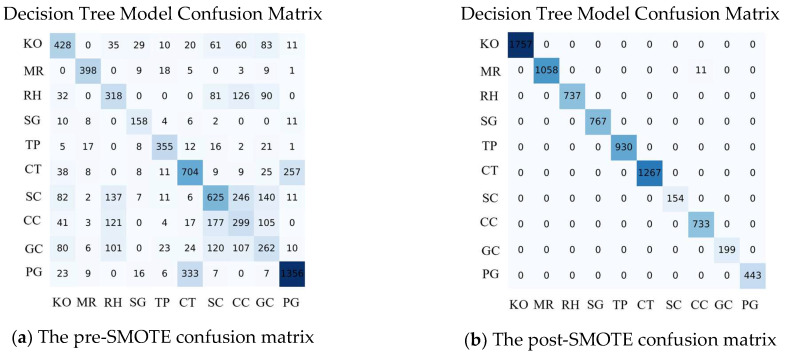
Decision Tree (DT) Model Confusion Matrix before and after applying SMOTE: (**a**) The pre-SMOTE confusion matrix highlights class imbalances, with higher misclassifications, particularly in underrepresented fish species. (**b**) The post-SMOTE confusion matrix demonstrates significant improvements in classification accuracy, reducing false positives and false negatives across all species. These results emphasize the effectiveness of SMOTE in addressing class imbalance, thereby enhancing the model’s predictive performance in multi-species aquaculture environments.

We evaluated 11 machine learning algorithms to assess their performance within the smart fish farming decision-making system, fine-tuning each model using GridSearch and SMOTE for optimal results. The performance evaluation highlighted significant variations across key metrics, including accuracy, precision, recall, F1-score, and Matthews Correlation Coefficient (MCC). The fine-tuned hyperparameters and the confusion matrix for the proposed Decision Tree (DT) model, both before and after the SMOTE application, are presented in Table 2 and Figure 6, respectively (For further details of results, see Supplementary Material.)

Among all the models, quantitative results highlighted the DT model’s excellence, achieving near-perfect metrics: an accuracy of 99.86%, a precision of 99.87%, a recall of 99.86%, an F1-score of 99.86%, and an MCC of 99.84%, as depicted in Table 3. This indicates a highly effective model with minimal misclassification, making it ideal for real-time, high-stakes decision-making in fish farming.

In contrast, the ensemble model (a combination of several models) delivered solid results with a 97.31% accuracy and matching values for precision, recall, and F1-score, but with a slightly lower MCC of 96.90%. Though it performed well, it did not surpass DT’s dominance in overall accuracy and robustness. Random Forest (RF), a popular ensemble technique, showed an accuracy of 94.16% and a precision and recall near 94.2%, indicating strong performance, but it lagged behind DT in both predictive accuracy and MCC (93.01%).

Similarly, K-Nearest Neighbors (KNN) and Bagging models exhibited comparable results, with accuracies around 94% and MCC values close to 93%, making them viable, though slightly less optimal options. On the lower end of the performance, Support Vector Machine (SVM) and Logistic Regression (LR) models significantly underperformed compared to DT. SVM achieved an accuracy of 85.78%, with a precision of 88.54%, while LR lagged with only a 57% accuracy and a much lower MCC of 51.61%. These results suggest that these models struggle with the complexities of the dataset. Interestingly, Stacking and Boosting models, which often outperform single classifiers, reached accuracies in the mid-90% range (94.64% for stacking and 94.10% for boosting), but neither could surpass the DT’s dominance. Finally, the Single-Layer Perceptron (SLP) and Multilayer Perceptron (MLP) neural network models also showed competitive results, with MLP achieving a 94% accuracy and SLP at 86.05%.

Overall, the Decision Tree model clearly outperforms other algorithms, achieving the highest accuracy, precision, recall, F1-score, and MCC, making it the most suitable choice for the intelligent decision-making system in this context. Other models like ensemble-based approaches (Boosting, Stacking, and RF) also performed well but fell short of DT’s near-perfect performance.

**Figure 7:**
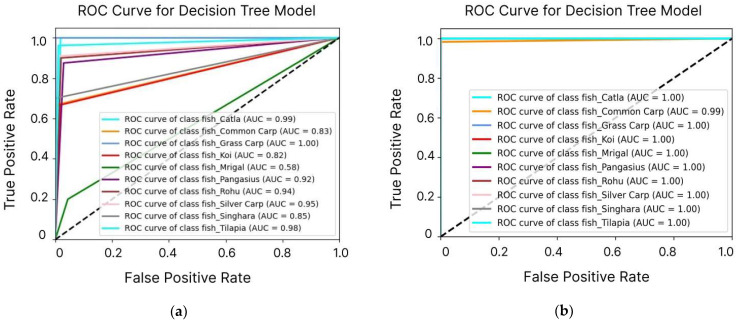
ROC curves of the DT model before and after SMOTE application: The dotted line represents the baseline performance of a random classifier (AUC = 0.5), against which the model’s performance is compared. (**a**) The model shows moderate discriminative ability and limitations in classification accuracy. (**b**) The model demonstrates a significant improvement in the AUC, indicating enhanced sensitivity and specificity, highlighting SMOTE’s effectiveness in optimizing the model’s performance for multi-class fish species prediction.

Figure 7 illustrates the ROC curves for the DT model used in predicting fish species survival. The first curve shows the model’s performance before the application of SMOTE, indicating a moderate ability to discriminate between classes, with some misclassifications, particularly in minority species. In contrast, the second curve displays the model’s performance after applying SMOTE, revealing a significant improvement in the AUC. This enhancement reflects increased sensitivity and specificity, demonstrating the effectiveness of SMOTE in addressing class imbalance and improving the model’s overall predictive accuracy. The figure emphasizes how data preprocessing techniques can significantly enhance a model’s ability to distinguish between different fish species’ survival conditions. The analysis also validated that the model is highly efficient in terms of speed, achieving top-tier predictive accuracy, especially in complex scenarios requiring class balancing.

### 3.2. Environmental Parameter Analysis for Optimal Fish Farming

We analyzed six ponds for aquaculture suitability based on three key environmental parameters. The recorded pH levels across the ponds were as follows: Pond 1 (6.40–8.50), Pond 2 (6.10–8.20), Pond 3 (10.00–14.00), Pond 4 (6.00–8.40), Pond 5 (10.00–14.00), and Pond 6 (6.30–8.30). Pond 1 falls squarely within the optimal pH range for fish farming (6.5–8.5), making it ideal for aquaculture. Pond 2 is slightly below this range but remains a viable option. In contrast, Ponds 3 and 5 exhibit excessively high pH levels.

Temperature readings across all ponds ranged from 18.00 to 21.00 °C, which is within the optimal range for aquaculture (17–25 °C). Turbidity levels in all ponds varied between 3 and 16 NTU across the ponds. While Ponds 1, 3, and 5 exhibited moderate turbidity, Ponds 2, 4, and 6 remained within the ideal range.

Overall, Ponds 1, 2, 4, and 6 are suitable for fish farming, while Ponds 3 and 5 are not, primarily due to their high pH levels. Pond 4 is marginally acceptable, while Pond 6 is within the ideal range. Maintaining proper water quality in these ponds is crucial for promoting fish health and growth.

**Figure 8:**
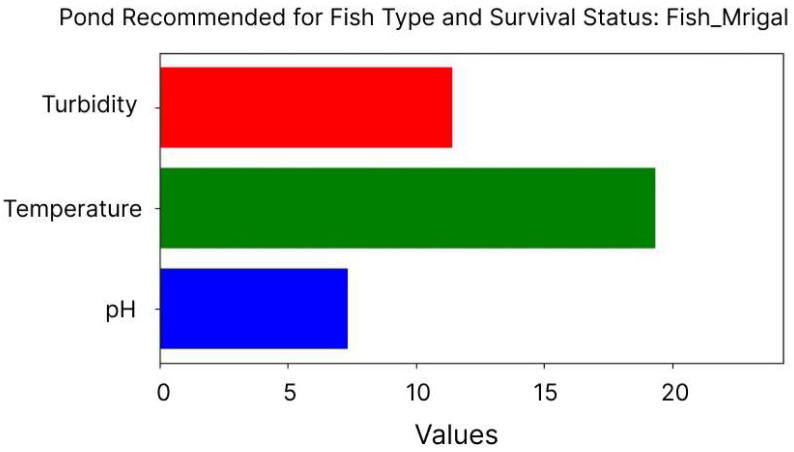
Real-time fish survival prediction based on water quality parameters.

Figure 8 illustrates the real-time prediction process for the survival of Mrigal fish species, based on water quality parameters collected from Pond 1 through an IoT-driven aquaculture system. The system recorded a pH of 7.2, a temperature of 18 °C, and a turbidity level of 11.5 NTU. Although the turbidity level is slightly elevated compared to the ideal range, Mrigal is known to tolerate higher turbidity without significant physiological stress. The model processes these parameters and predicts that Mrigal can survive under these conditions, demonstrating the system’s capacity to adapt its predictions to species-specific tolerances. The figure likely demonstrates how real-time data are analyzed, and the model provides immediate feedback on water conditions and fish survival predictions. Such capabilities enable fish farmers to optimize environmental management strategies, improving species survival and overall productivity.

Our model surpasses existing methods in accuracy and cost-effectiveness. By minimizing labor demands and enhancing scalability across multiple ponds, the system offers a flexible, robust solution for aquaculture management, adaptable to various fish farming contexts.

### 3.3. Model Validation and Generalization Across Multi-Regional Datasets

The model was rigorously tested on both publicly available datasets and our custom-built dataset from different regions, including Bangladesh and Pakistan. Its performance is evaluated using key metrics such as accuracy, precision, recall, F1-score, and MCC. The results demonstrated consistently high accuracy—94.57%, 96.19%, and 99.86%—as summarized in Table 4, affirming the model’s robustness across diverse environmental conditions and species.

**Figure 9:**
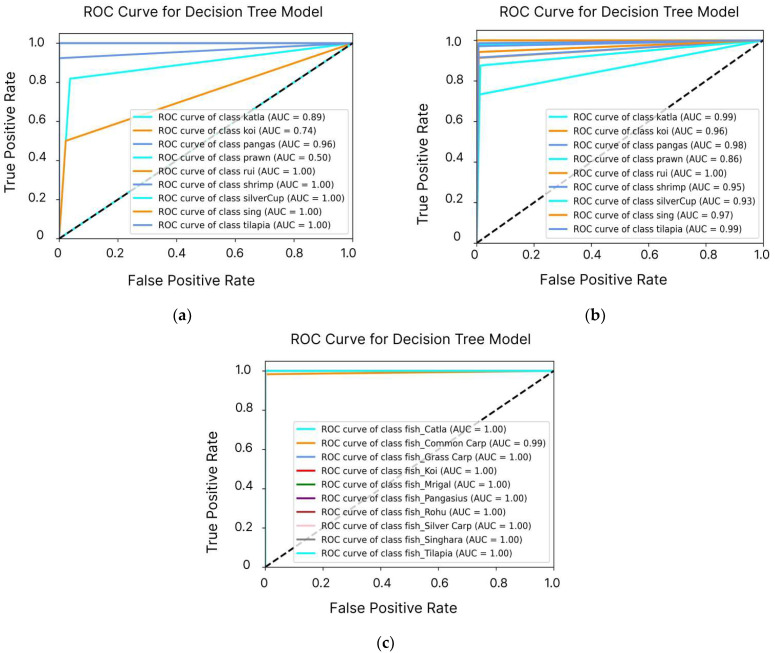
ROC curves of the optimal DT model, evaluated on three datasets: (**a**) Dataset-1, (**b**) Dataset-2, and (**c**) custom-built dataset. The dotted line represents the baseline performance of a random classifier (AUC = 0.5), against which the model’s performance is compared. AUC values and evaluation metrics such as accuracy, precision, recall, and F1-score illustrate the model’s high performance and generalizability across different regions and datasets.

The ROC curves, illustrated in Figure 9, further highlight the model’s classification performance. Dataset-1 showed moderate AUC values, while Dataset-2 showed improved performance, with most fish species achieving higher AUCs. The custom-built dataset, in particular, demonstrated near-perfect AUC values, indicating exceptional classification accuracy for all species.

The model’s ability to capture and leverage the unique characteristics of different fish species ensures precise classification across datasets, validating the robustness and generalizability of our model across diverse data sources and ensuring that our findings are not dataset-specific but rather applicable in varied aquaculture contexts. This high degree of generalizability guarantees that the model can reliably be applied to new datasets in similar aquaculture settings, making it a robust tool for intelligent decision-making in fish farming systems.

## 4. Conclusions

This study introduces a novel IoT-driven system for real-time water quality monitoring, marking a significant advancement in climate-smart aquaculture management. A key innovation lies in the comprehensive dataset collected over 157 days from six diverse ponds across various regions, capturing key water quality parameters such as pH, temperature, and turbidity, critical variables that are increasingly affected by climate variability. Advanced data cleaning techniques—including feature scaling, outlier, random noise detection, and SMOTE for class balancing—were applied to enhance data quality and reliability, paving the way for climate-adaptive predictions. In addition to robust dataset preparation, we employed hyperparameter tuning and model optimization techniques, using Grid Search to fine-tune the model parameters for the best performance. Through comprehensive model selection, the Decision Tree model emerged as the most effective, achieving outstanding performance metrics, including 99.86% accuracy, 99.87% precision, and 99.84% Matthews Correlation Coefficient (MCC). These metrics, validated across multiple datasets, underscore the resilience and generalizability of the model in diverse aquaculture settings.

Our findings not only enhance the operational efficiency and sustainability of aquaculture practices but also introduce a scalable framework adaptable to various geographic and climate conditions. The integration of cloud-based real-time monitoring with predictive analytics offers proactive management of water quality, fostering healthier aquatic ecosystems while optimizing resource allocation. This system helps fish farming operations become more resilient to climate-induced water quality fluctuations, contributing to improved fish survival and profitability.

While the cost-effective IoT-driven intelligent decision-making system has demonstrated exceptional potential in real-time water quality monitoring and optimizing conditions for multi-species aquaculture, the hardware setup does have some limitations. Notably, the system’s components have a lifespan of 3 to 5 years and require periodic maintenance to ensure optimal performance. To address these limitations, future improvements could involve integrating advanced sensors for additional parameters, such as dissolved oxygen and ammonia, and utilizing more powerful microcontrollers to enhance scalability. Moving forward, efforts will focus on extending the system’s capabilities by incorporating additional environmental parameters and automating water quality management, contributing to the advancement of sustainable, climate-resilient aquaculture practices globally.

## Figures and Tables

**Table 1:** Standard reference values for water quality parameters.

Water Quality Parameter	Reference Value
Temperature	25–32 °C, or >20 °C
pH	6.5–8.5
Turbidity	0–25 NTU
Dissolved Oxygen (DO)	>5 mg/L
Biochemical Oxygen Demand (BOD	<5 mg/L
Total Dissolved Solids (TDS)	400 mg/L
Chemical Oxygen Demand (COD)	20–30 mg/L
Total Suspended Solids (TSS)	<80 mg/L
Electrical Conductivity (EC)	150–500 µS/cm
Hardness	>15 mg/L
Alkalinity	50–300 mg/L
Nitrite (NO₂)	<0.2 mg/L
Nitrate (NO₃)	0–100 mg/L
Total Ammonia Nitrogen (TAN)	0–0.2 mg/L

**Table 2:** Fine-tuned parameters for enhanced DT performance in fishpond monitoring.

Hyperparameter	Criterion	Max_Depth	Min_Samples Leaf	Min_Samples Split
**Value**	entropy	5	1	2

**Table 3:** Evaluation of predictive models for real-time fish survival monitoring.

ML-Models	ACC	Pre	Rec	F1	MCC
DT	0.9986	0.9987	0.9986	0.9986	0.9984
Ensemble	0.9731	0.9731	0.9731	0.9731	0.9690
RF	0.9416	0.9420	0.9416	0.9417	0.9301
SVM	0.8578	0.8854	0.8578	0.8655	0.8333
KNN	0.9375	0.9388	0.9375	0.9378	0.9256
LR	0.5700	0.6953	0.5700	0.5949	0.5161
Bagging	0.9418	0.9418	0.9418	0.9419	0.9419
Boosting	0.9410	0.9438	0.9410	0.9417	0.9298
Stacking	0.9464	0.9473	0.9464	0.9466	0.9358
SLP	0.8605	0.8784	0.8605	0.8664	0.8350

**Table 4:** Comprehensive evaluation results of the DT model across multi-regional datasets for fish survival in different pond setups.

Evaluation Results on Test Datasets	ACC	Pre	Rec	F1	MCC
Dataset-1 [26]	0.9457	0.9447	0.9457	0.9444	0.9349
Dataset-2 [27]	0.9619	0.9620	0.9619	0.9619	0.9546
Custom-built dataset	0.9986	0.9987	0.9986	0.9986	0.9984

## Data Availability

The dataset supporting the findings of this study is not publicly available. However, it can be accessed upon reasonable request from the corresponding author.

## Supplementary Materials

The following supporting information can be downloaded at: https://www.mdpi.com/article/10.3390/s24237842/s1, The ROC curves and confusion matrix before applying SMOTE and after applying SMOTE for all the models; Table S1: Result Evaluation of Predictive Models for Real-Time Fish Survival Monitoring.
