# Supplementary material for: An Optimal Internet of Things-Driven Intelligent Decision-Making System for Real-Time Fishpond Water Quality Monitoring and Species Survival
Source: Sensors (Basel). 2024 Dec 8;24(23):7842. doi: 10.3390/s24237842 (PMC11644897; doi:10.3390/s24237842)
Supplement: Supplementary file 1 [file sensors-24-07842-s001.zip › sensors-3275673-supplementary.pdf]

An Optimal Internet of Things-Driven Intelligent Decision-Making System for Real-Time Fishpond Water Quality Monitoring and Species Survival

Saima Kanwal [1,2†], Muhammad Abdullah [3†], Sahil Kumar [4], Saqib Arshad [2], Muhammad Shahroz [5], Dawei Zhang[1], and Dileep Kumar [5,*]

[1] Engineering Research Centre of Optical Instrument and Systems, Ministry of Education and Shanghai Key Lab of Modern Optical System, University of Shanghai for Science and Technology, No. 516 Jun Gong Road, Shanghai 200093, China
[2] Department of Biomedical Engineering, School of Health Science and Engineering, University of Shanghai for Science and Technology, No. 516 Jun Gong Road, Shanghai 200093, China
[3] Faculty of Computing, The Islamia University of Bahawalpur, Bahawalpur 63100, Punjab, Pakistan
[4] Department of Software Engineering, Shaheed Zulfikar Ali Bhutto Institute of Science and Technology (SZABIST), Karachi 75600, Pakistan
[5] Faculty of Engineering, The Islamia University of Bahawalpur, Bahawalpur 63100, Punjab, Pakistan

† These authors contributed equally
* Correspondence: dileep.kumar@iub.edu.pk

Table S1. Result Evaluation of Predictive Models for Real-Time Fish Survival Monitoring

ML-Models	ACC	Pre	Rec	F1	MCC
DT	0.9986	0.9987	0.9986	0.9986	0.9984
Ensemble	0.9731	0.9731	0.9731	0.9731	0.9690
RF	0.9416	0.9420	0.9416	0.9417	0.9301
SVM	0.8578	0.8854	0.8578	0.8655	0.8333
KNN	0.9375	0.9388	0.9375	0.9378	0.9256
LR	0.5700	0.6953	0.5700	0.5949	0.5161
Bagging	0.9418	0.9418	0.9418	0.9419	0.9419
Boosting	0.9410	0.9438	0.9410	0.9417	0.9298
Stacking	0.9464	0.9473	0.9464	0.9466	0.9358
SLP	0.8605	0.8784	0.8605	0.8664	0.8350

The initial ROC curves and confusion matrix, shown in the figures below, represent the models' performance before applying SMOTE. These results indicate a moderate ability to distinguish between fish species, with noticeable misclassifications, particularly in the

minority species. However, after applying SMOTE, the second set of ROC curves and confusion matrix demonstrates a substantial improvement in model performance. Specifically, there is a significant increase in the AUC scores, highlighting the model's enhanced capacity to correctly classify fish species, especially those in underrepresented classes.

DT Model

Hyperparameter	criterion	max_depth	min_samples_leaf	min_samples_split
Value	entropy	5	1	2

```
Decision Tree  Results:
Accuracy: 0.7755
Precision: 0.7773
Recall: 0.7755
F1 Score: 0.7741
MCC: 0.7422
```

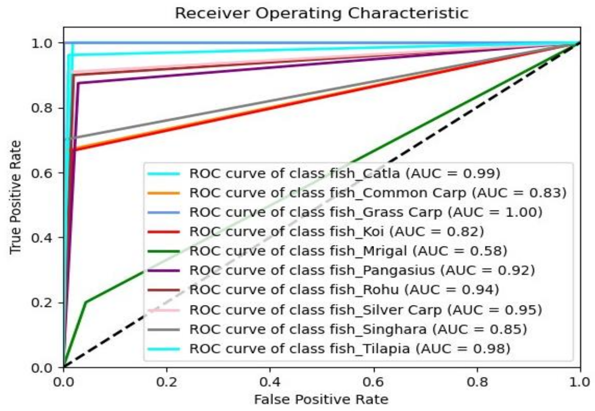

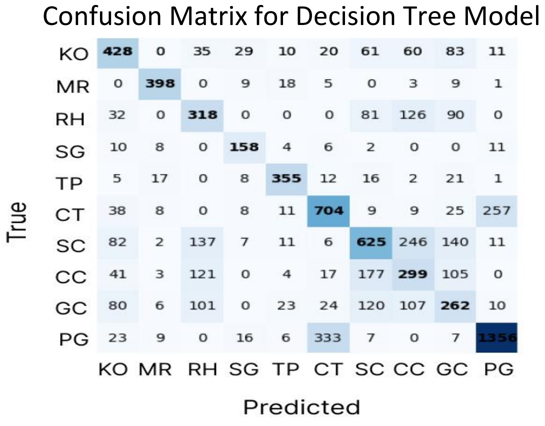

Before SMOTE

```
Decision Tree Results:
Accuracy: 0.9986
Precision: 0.9987
Recall: 0.9986
F1 Score: 0.9986
MCC: 0.9984
```

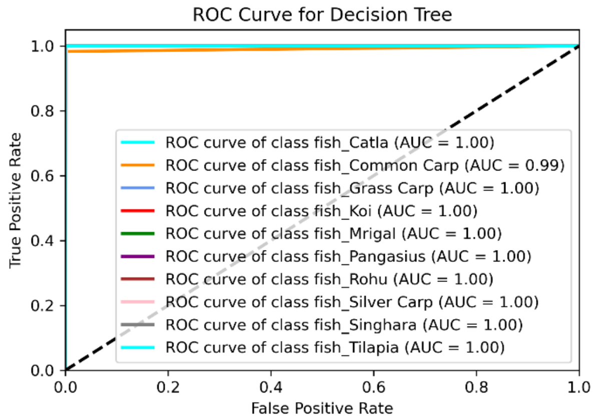

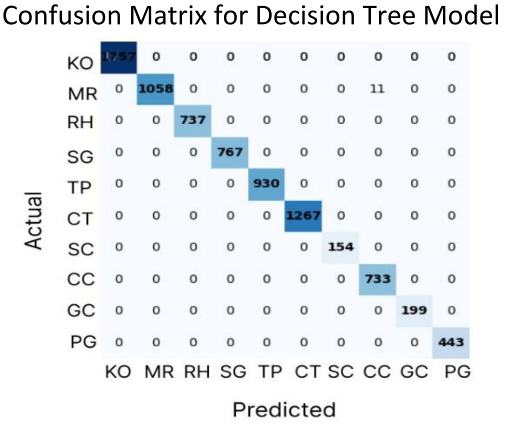

After SMOTE

Ensemble Model

Hyperparameter	Voting Scheme	Boosting	Bagging	Stacking
Value	soft	(XGBoost)	DT, KNN	SVM, RF

Custom Ensemble Model Results:

Accuracy: 0.7218

Precision: 0.7218

Recall: 0.7218

F1 Score: 0.7177

MCC: 0.6806

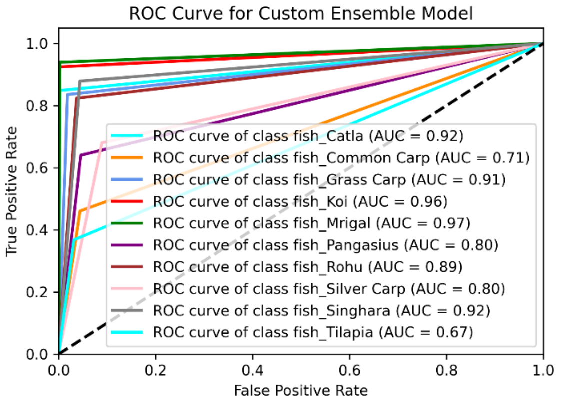

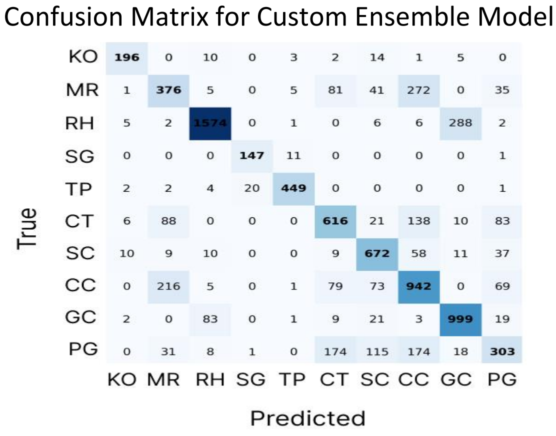

Before SMOTE

Custom Ensemble Model Results:

Accuracy: 0.9731

Precision: 0.9731

Recall: 0.9731

F1 Score: 0.9731

MCC: 0.9690

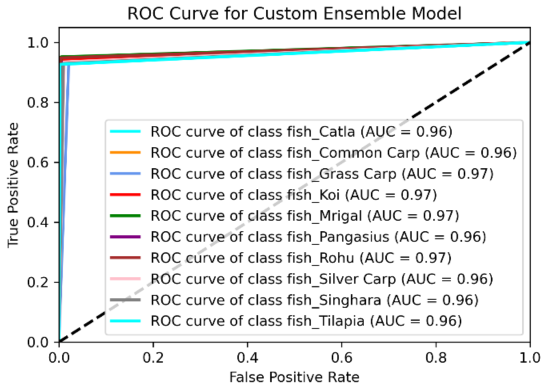

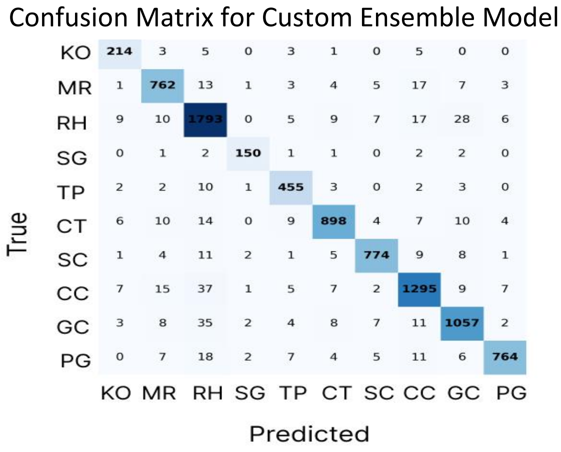

After SMOTE

RF Model

Hyperparameter	criterion	max_depth	n_estimators	min_samples_split
Value	entropy	4	100	2

Random Forest Model Results:

Accuracy: 0.7141

Precision: 0.7161

Recall: 0.7141

F1 Score: 0.7126

MCC: 0.6715

Random Forest Results:

Accuracy: 0.9416

Precision: 0.9420

Recall: 0.9416

F1 Score: 0.9417

MCC: 0.9301

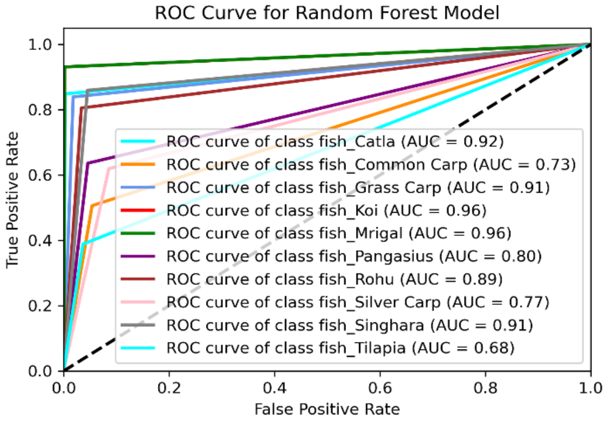

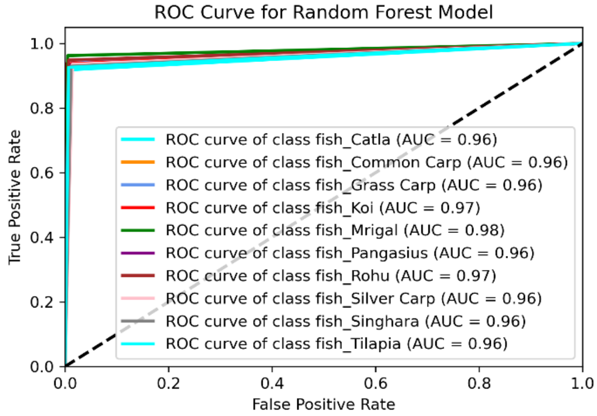

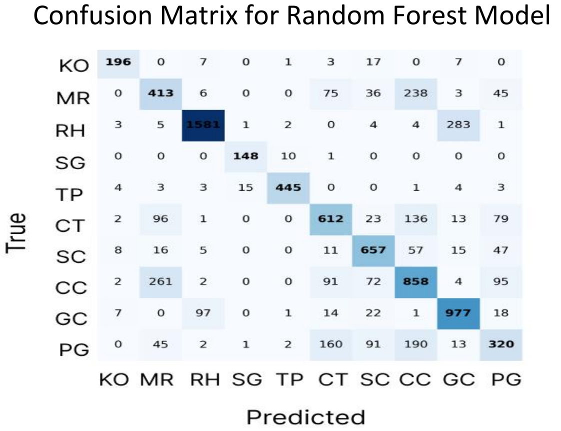

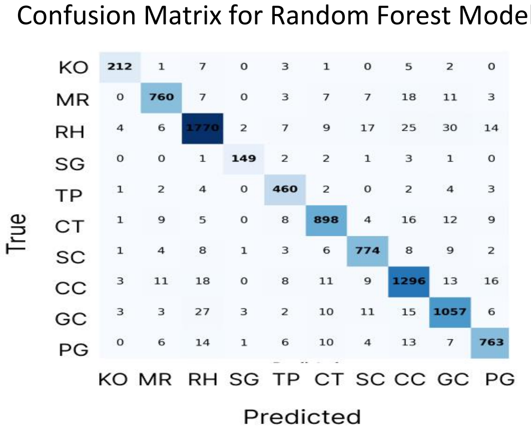

Before SMOTE **After SMOTE**

SVM Model

Hyperparameter	C (Regularization)	Kernel	Gamma	Degree
Value	1	rbf	scale	3

Stacking Model Results:
Accuracy: 0.7117
Precision: 0.7124
Recall: 0.7117
F1 Score: 0.7095
MCC: 0.6685

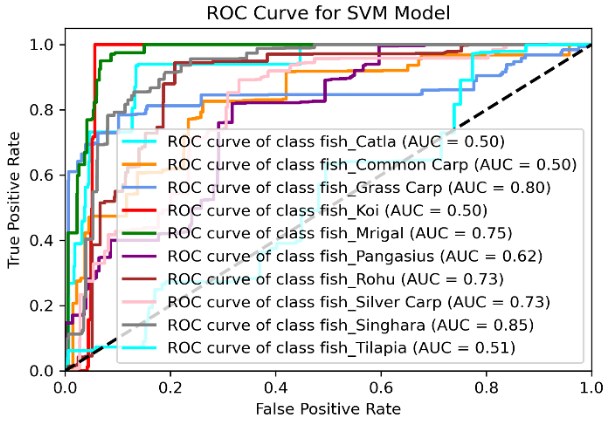

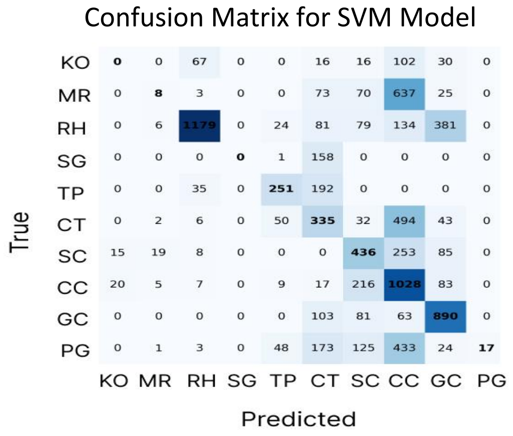

Before SMOTE

SVM Results:
Accuracy: 0.8578
Precision: 0.8854
Recall: 0.8578
F1 Score: 0.8655
MCC: 0.8333

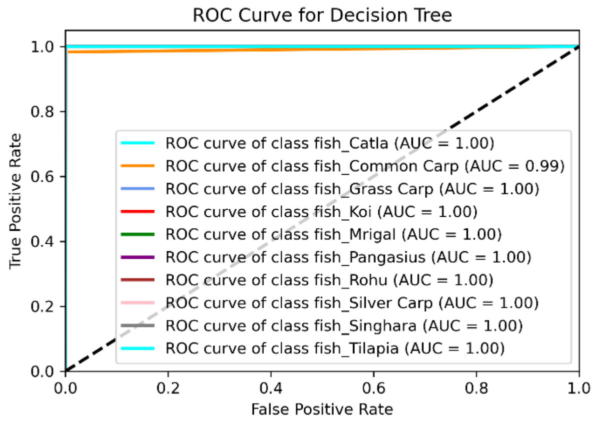

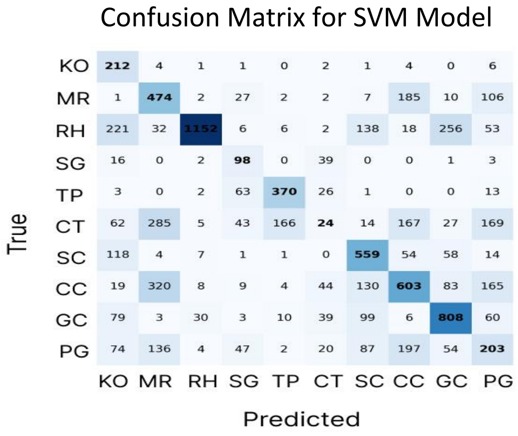

After SMOTE

KNN Model

Hyperparameter	n_neighbors	Weight Function	Distance Metric	Algorithm
Value	4	uniform	Euclidean	auto

KNN Results:
Accuracy: 0.6878
Precision: 0.6888
Recall: 0.6878
F1 Score: 0.6856
MCC: 0.6408

K-Nearest Neighbors Results:
Accuracy: 0.9375
Precision: 0.9388
Recall: 0.9375
F1 Score: 0.9378
MCC: 0.9256

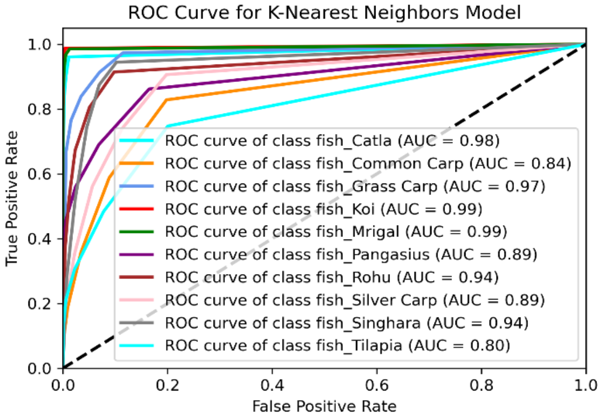

Before SMOTE

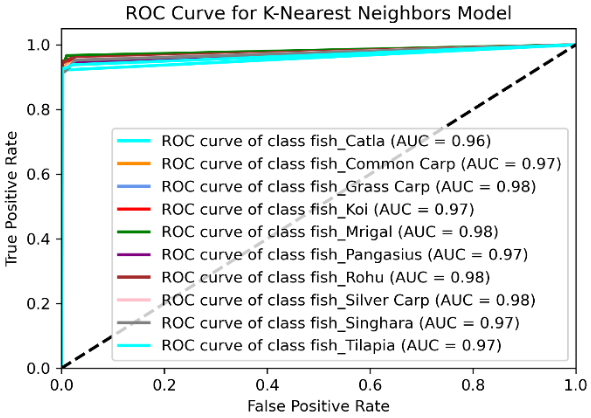

After SMOTE

LR Model

Hyperparameter	Solver	C (Regularization)	Penalty	Max Iteration
Value	lbfgs	1	12	100

Logistic Regression Model Results:

Accuracy: 0.4053

Precision: 0.3747

Recall: 0.4053

F1 Score: 0.3695

MCC: 0.3048

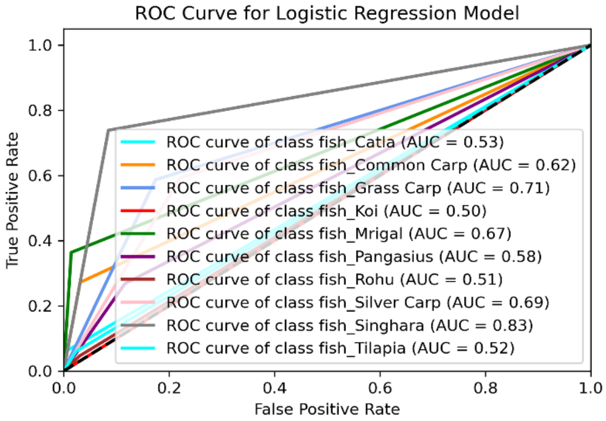

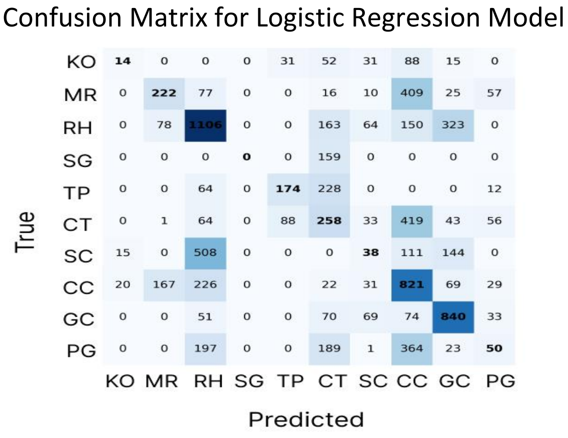

Before SMOTE

Logistic Regression Results:

Accuracy: 0.5700

Precision: 0.6953

Recall: 0.5700

F1 Score: 0.5949

MCC: 0.5161

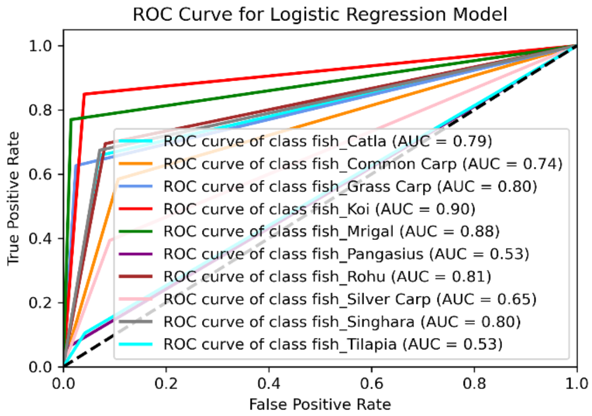

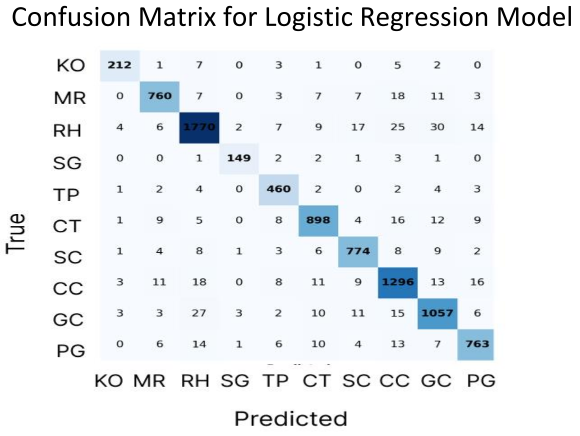

After SMOTE

Bagging

Hyperparameter	n_estimators	max_depth	Max Features	Bootstrap
Value	100	4	sqrt	True

Bagging Model Results:
Accuracy: 0.7024
Precision: 0.7023
Recall: 0.7024
F1 Score: 0.7014
MCC: 0.6575

Bagging Results:
Accuracy: 0.9418
Precision: 0.9423
Recall: 0.9418
F1 Score: 0.9419
MCC: 0.9304

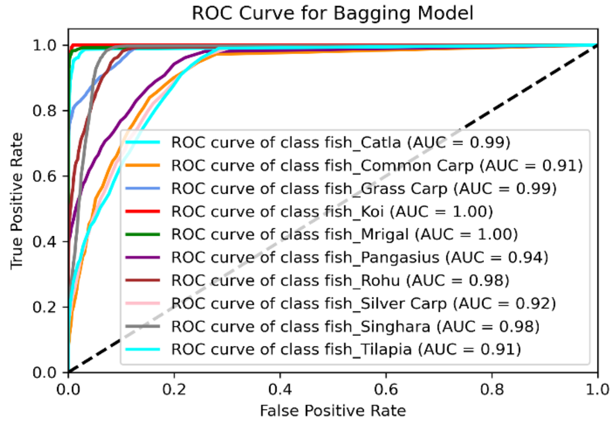

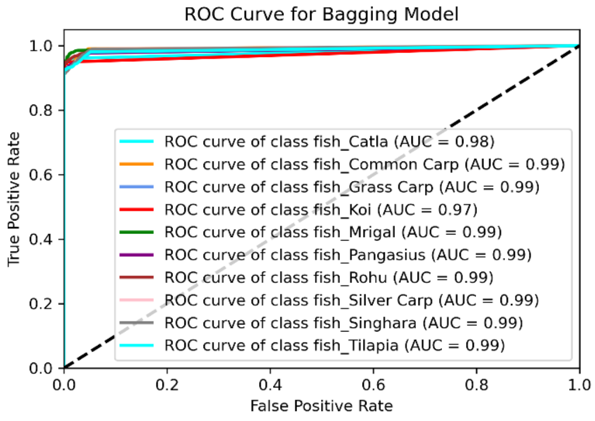

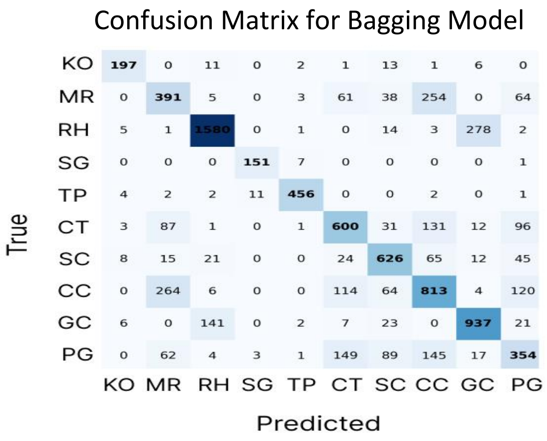

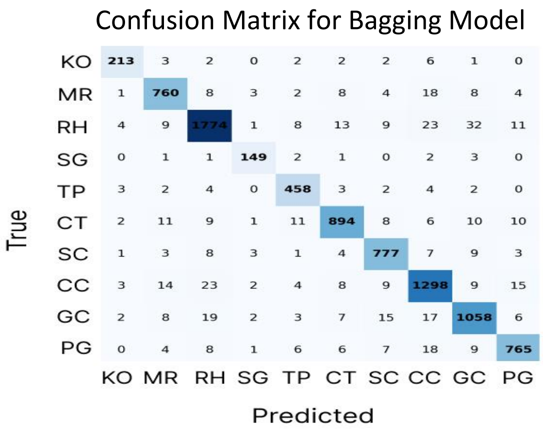

Before SMOTE **After SMOTE**

Boosting

Hyperparameter	Learning Rate	max_depth	n_estimators	Subsample
Value	0.001	4	100	0.8

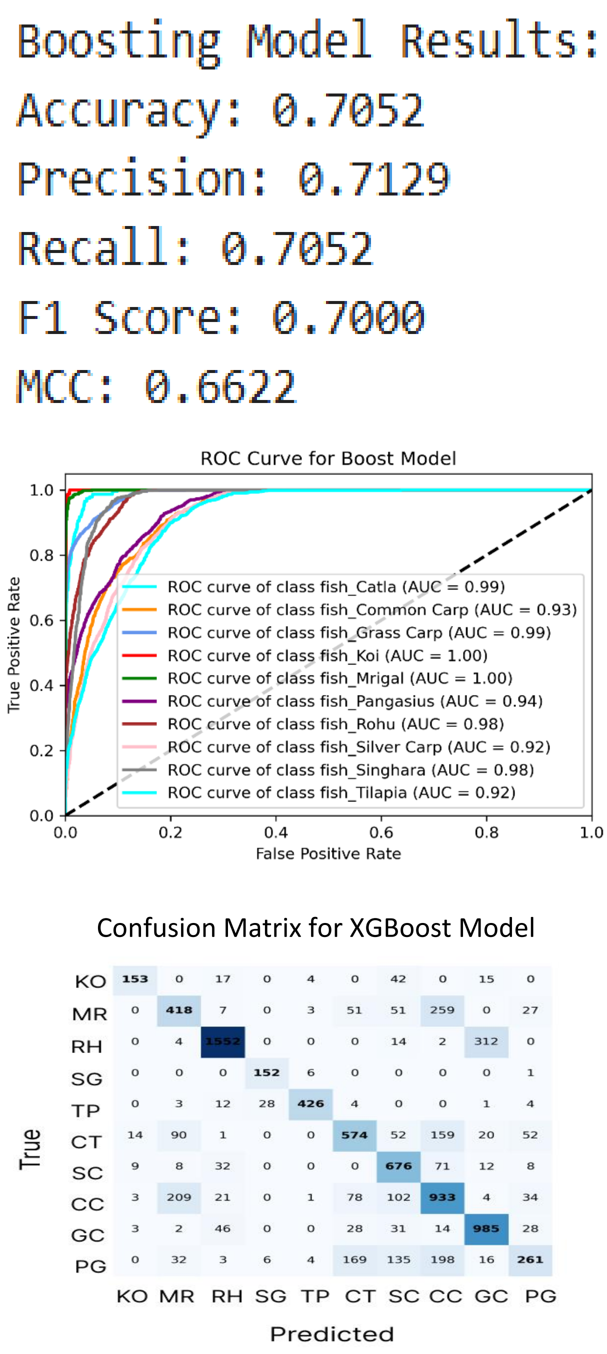

Before SMOTE

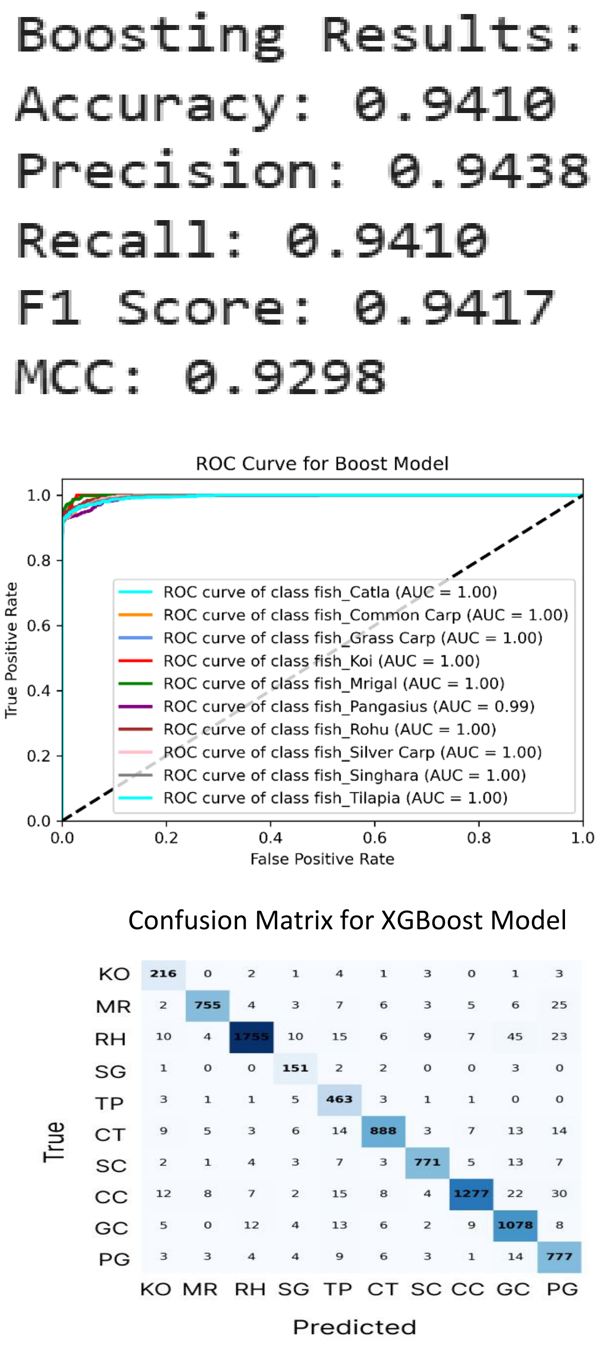

After SMOTE

Stacking

Hyperparameter	Base Models	Meta Model	Cross Validation Folds	Use Proba (Meta)
Value	RF, LR, KNN	Logistic Regression	5	True

Stacking Model Results:
Accuracy: 0.7117
Precision: 0.7124
Recall: 0.7117
F1 Score: 0.7095
MCC: 0.6685

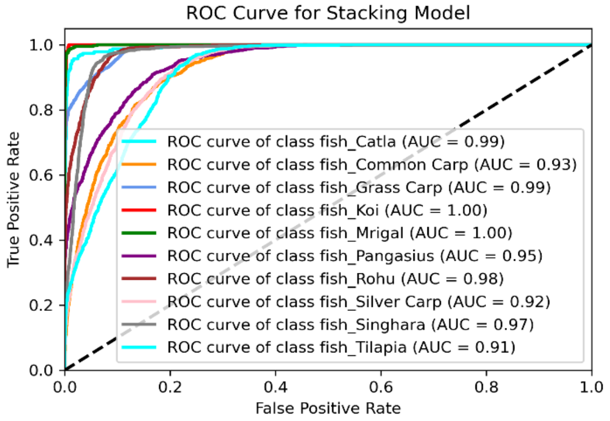

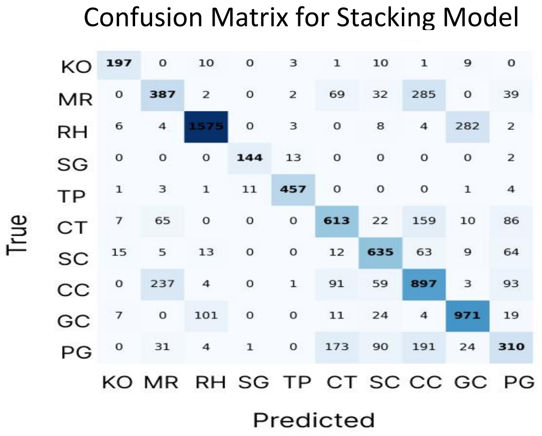

Before SMOTE

Stacking Results:
Accuracy: 0.9464
Precision: 0.9473
Recall: 0.9464
F1 Score: 0.9466
MCC: 0.9358

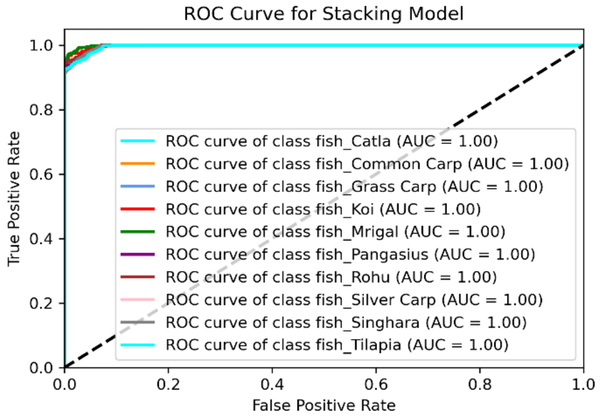

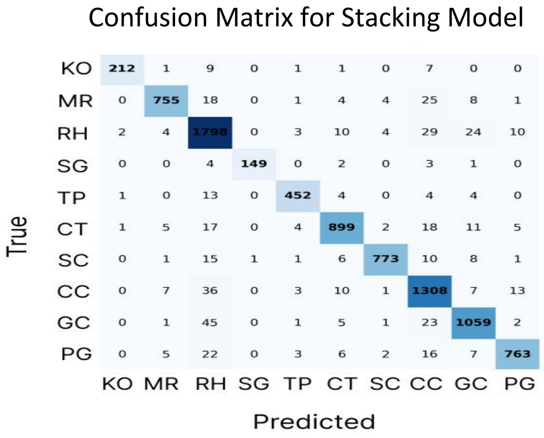

After SMOTE

SLP (Single-Layer Perceptron) Model

Hyperparameter	Learning Rate	Max Iterations	Activation Function	Solver
Value	0.001	200	ReLU	Adam

```
SLP Model Results:
Accuracy: 0.4100
Precision: 0.3823
Recall: 0.4100
F1 Score: 0.3711
MCC: 0.3110
```

Confusion Matrix for SLP Model

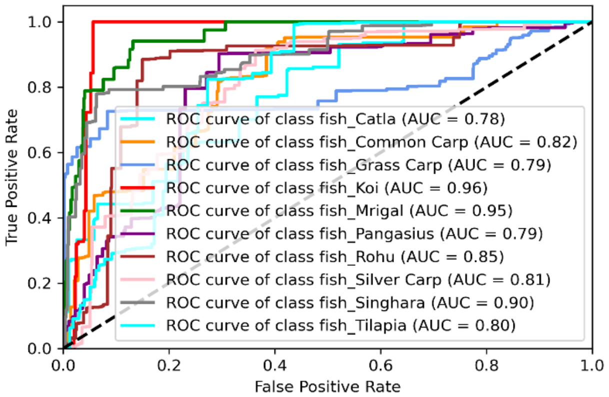

Confusion Matrix for SLP Model

True \ Predicted	KO	MR	RH	SG	TP	CT	SC	CC	GC	PG
KO	**14**	0	0	0	31	52	31	88	15	0
MR	0	**170**	76	0	0	17	10	462	25	56
RH	0	78	**1105**	0	0	163	64	150	324	0
SG	0	0	0	**0**	0	159	0	0	0	0
TP	0	0	64	0	**174**	228	0	0	0	12
CT	0	0	64	0	77	**280**	33	420	43	45
SC	15	0	508	0	0	0	**38**	111	144	0
CC	20	110	226	0	0	22	44	**850**	70	43
GC	0	0	7	0	0	100	69	74	**884**	3
PG	0	0	198	0	0	190	1	363	23	**49**

Before SMOTE

```
Single Layer Perceptron Results:
Accuracy: 0.8605
Precision: 0.8784
Recall: 0.8605
F1 Score: 0.8664
MCC: 0.8350
```

Confusion Matrix for SLP Model

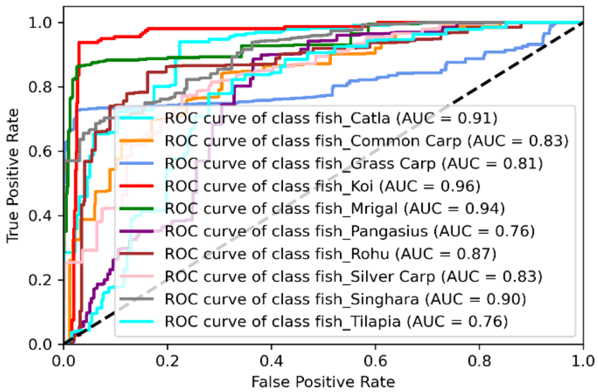

Confusion Matrix for SLP Model

True \ Predicted	KO	MR	RH	SG	TP	CT	SC	CC	GC	PG
KO	**152**	4	1	61	1	3	2	2	0	5
MR	25	**478**	11	25	3	16	44	144	24	46
RH	217	35	**1180**	0	2	8	80	19	316	27
SG	0	0	2	**135**	1	19	0	0	1	1
TP	1	2	7	62	**368**	28	1	0	0	9
CT	62	286	11	133	105	**63**	35	167	4	96
SC	117	4	16	0	1	3	**577**	53	38	7
CC	19	375	24	13	5	13	201	**545**	91	99
GC	63	2	52	0	1	5	191	6	**767**	50
PG	78	135	46	58	4	5	88	269	71	**70**

After SMOTE

MLP Model

Hyperparameter	Hidden Layers	Learning Rate	Activation Function	Batch Size
Value	(64, 32)	0.001	ReLU	32

```
MLP Model Results:
Accuracy: 0.7841
Precision: 0.7979
Recall: 0.7841
F1 Score: 0.7851
MCC: 0.7540
```

Confusion Matrix for Multilayer Perceptron Model

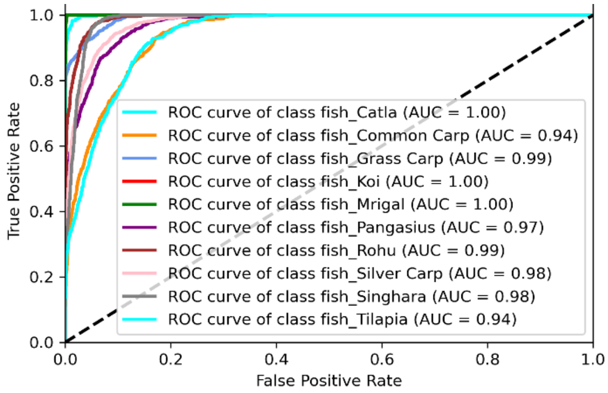

Confusion Matrix for Multilayer Perceptron Model

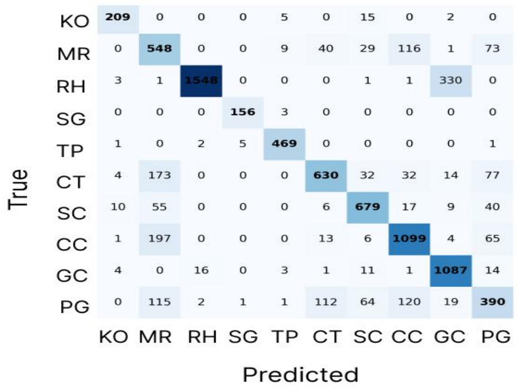

Before SMOTE

```
Multilayer Perceptron Results:
Accuracy: 0.9400
Precision: 0.9417
Recall: 0.9400
F1 Score: 0.9404
MCC: 0.9285
```

Confusion Matrix for Multilayer Perceptron Model

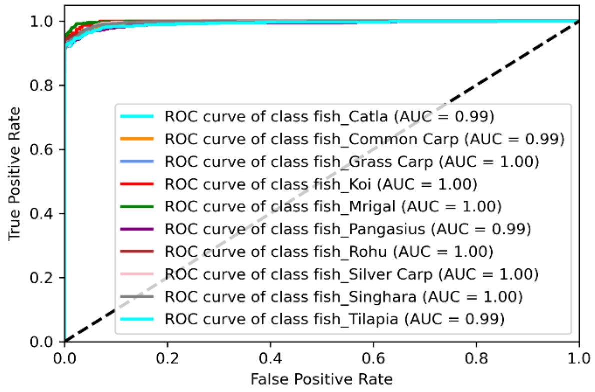

Confusion Matrix for Multilayer Perceptron Model

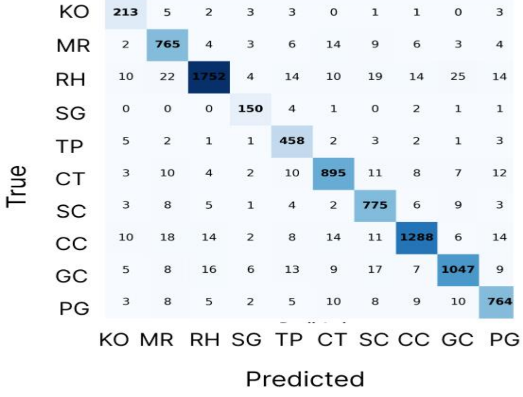

After SMOTE
